# Oligotrophic Growth of Nitrate-Dependent Fe^2+^-Oxidising Microorganisms Under Simulated Early Martian Conditions

**DOI:** 10.3389/fmicb.2022.800219

**Published:** 2022-03-28

**Authors:** Alex Price, Michael C. Macey, Victoria K. Pearson, Susanne P. Schwenzer, Nisha K. Ramkissoon, Karen Olsson-Francis

**Affiliations:** ^1^School of Environment, Earth and Ecosystem Sciences, Faculty of Science, Technology, Engineering, and Mathematics, The Open University, Milton Keynes, United Kingdom; ^2^School of Physical Sciences, Faculty of Science, Technology, Engineering, and Mathematics, The Open University, Milton Keynes, United Kingdom

**Keywords:** oligotrophy, iron, nitrate, Mars, NDFO, NRFeOx, simulant

## Abstract

Nitrate-dependent Fe^2+^ oxidation (NDFO) is a microbially mediated process observed in many anaerobic, low-nutrient (oligotrophic) neutral–alkaline environments on Earth, which describes oxidation of Fe^2+^ to Fe^3+^ in tandem with microbial nitrate reduction. Evidence suggests that similar environments existed on Mars during the Noachian epoch (4.1–3.7 Ga) and in periodic, localised environments more recently, indicating that NDFO metabolism could have played a role in a potential early martian biosphere. In this paper, three NDFO microorganisms, *Acidovorax* sp. strain BoFeN1, *Pseudogulbenkiania* sp. strain 2002 and *Paracoccus* sp. strain KS1, were assessed for their ability to grow oligotrophically in simulated martian brines and in a minimal medium with olivine as a solid Fe^2+^ source. These simulant-derived media were developed from modelled fluids based on the geochemistry of Mars sample locations at Rocknest (contemporary Mars soil), Paso Robles (sulphur-rich soil), Haematite Slope (haematite-rich soil) and a Shergottite meteorite (common basalt). The Shergottite medium was able to support growth of all three organisms, while the contemporary Mars medium supported growth of *Acidovorax* sp. strain BoFeN1 and *Pseudogulbenkiania* sp. strain 2002; however, growth was not accompanied by significant Fe^2+^ oxidation. Each of the strains was also able to grow in oligotrophic minimal media with olivine as the sole Fe^2+^ source. Biomineralised cells of *Pseudogulbenkiania* sp. strain 2002 were identified on the surface of the olivine, representing a potential biosignature for NDFO microorganisms in martian samples. The results suggest that NDFO microorganisms could have thrived in early martian groundwaters under oligotrophic conditions, depending on the local lithology. This can guide missions in identifying palaeoenvironments of interest for biosignature detection. Indeed, biomineralised cells identified on the olivine surface provide a previously unexplored mechanism for the preservation of morphological biosignatures in the martian geological record.

## Introduction

Oligotrophic environments comprise much of the microbial biosphere on Earth and include many locations where certain conditions can be considered analogous to extraterrestrial locations, such as Mars (Bonilla-Rosso et al., 2012; Schmidt et al., 2018; Parro et al., 2019). A wide variety of biogeochemical cycles operate in these oligotrophic ecosystems, meaning that investigation of the myriad microbial processes hosted here can yield insights into both the terrestrial biosphere and potential extinct or extant biospheres on Mars.

Owing to the Fe-rich composition of the martian surface (Bertka and Fei, 1998; Halliday et al., 2001; Papike, 2018) the feasibility of Fe biogeochemical cycling has been discussed previously (Nixon et al., 2012). Studies have predominantly focused on aerobic Fe oxidation and organotrophic Fe reduction as plausible metabolisms on Mars (Amils et al., 2011; Popa et al., 2012; Bauermeister et al., 2014; Nixon, 2014). However, the detection of nitrates (70–1,100 ppm) in mudstone deposits at Gale crater (a circumneutral palaeolake environment; Stern et al., 2015) and from the meteorite EETA79001 (Kounaves et al., 2014), together with the confirmation of complex organics (Eigenbrode et al., 2018), have expanded the list of plausible electron donors and acceptors on Mars to include reduced organic compounds and oxidised nitrogen species.

Nitrate-dependent Fe^2+^ oxidation (NDFO) metabolism—also known as nitrate-reducing Fe oxidation (NRFeOx)—was recognised as a geobiological process over two decades ago in anoxic soils, waters and sediments (Hafenbradl et al., 1996; Straub et al., 1996; Benz et al., 1998). Although the biochemical mechanisms are yet to be fully resolved, isolates and enrichment cultures have been identified that couple Fe^2+^ oxidation with nitrate reduction during apparent autotrophic and mixotrophic growth, as well as Fe^2+^ oxidation caused by organotrophic denitrification (Straub et al., 1996; Weber et al., 2006b; Muehe et al., 2009; Carlson et al., 2013). Some claims of autotrophic growth by NDFO organisms are controversial [see discussion in Bryce et al., 2018]. One reason is that results could be obfuscated by the potential for both the carry-over of internal carbon sources within washed cells or by low-level residual organic carbon within batch culture apparatus and media components.

Circumneutral–alkaline conditions, such as those described for lacustrine environments on early Mars (Grotzinger et al., 2014), are required for NDFO to be an energy-yielding process (Weber et al., 2006a). NDFO provides less energy (−481.15 kJ mol^−1^ NO^3−^) than both organotrophic denitrification (−556 kJ mol^−1^ NO^3−^) and organotrophic nitrate ammonification (−623 kJ mol^−1^ NO^3−^; Strohm et al., 2007). However, the process is exergonic at circumneutral pH (ΔG°′ = −481.15 kJ mol^−1^ NO^3−^, −96.23 kJ mol^−1^ Fe) and, theoretically, provides enough energy to sustain growth under mixotrophic (Muehe et al., 2009; Weber et al., 2009) or autotrophic conditions, although this would require 26 moles Fe^2+^ to fix 1 mole C (Laufer et al., 2016; Bryce et al., 2018). NDFO could, therefore, have represented an important microbial process under circumneutral, near-surface conditions on early Mars (Price et al., 2018). However, most experiments using these organisms have used high Fe^2+^ (10 mM) that are not representative of typical oligotrophic aqueous environments on early Mars (Weber et al., 2006b; Miot et al., 2009a).

To address the plausibility of NDFO as a potential metabolism under oligotrophic early martian conditions, simulation experiments are required. Prior laboratory-based work has assessed the habitability of Mars across a range of chemical, pH, redox and physical conditions (Allen et al., 1997; Peters et al., 2008; Böttger et al., 2012; Stevens et al., 2018). Various brine-derived media have been developed to recreate aqueous environments representative of different martian locations and historical periods. For example, Fox-Powell et al. (2016) utilised the thermodynamically modelled brines (derived from weathering of a generalised synthetic martian basalt) of Tosca et al. (2011) to develop growth media, which could be used to study the impact of ionic strength on habitability. Despite the contribution made to our understanding of martian fluid chemistry and habitability, these brines were not suitable for this investigation for two reasons. Firstly, the ionic concentrations of these highly saline brines are intended to represent the evaporative conditions of the Hesperian era, rather than the hydrologically active Noachian period that carries the most interest in terms of NDFO and Fe^2+^ oxidising metabolisms more generally on Mars. Secondly, the modelled brines assumed that all Fe is present entirely in the ferrous form (Fe^2+^), which could be unrealistic when considering the variability in Fe oxidation state across martian environments and have a bearing on the viability of an Fe^2+^-oxidising metabolism.

To address these issues and create a link to *in situ* data, we have developed martian brines based on a suite of simulants (Ramkissoon et al., 2019). These simulants themselves are based on the geochemical composition of four different martian lithologies: (1) the sulphur-rich (SR) Paso Robles regolith found at Columbia Hills, Gusev crater; (2) a haematite-rich (HR) deposit discovered at Meridiani Planum (Rieder et al., 2004; Lane et al., 2008); (3) the Rocknest regolith at Gale crater (Blake et al., 2013) as a contemporary Mars (CM) simulant due to similarity to the global Mars mean regolith composition (Ramkissoon et al., 2019); and (4) a Shergottite (SG) meteorite (Bridges and Warren, 2006), representative of the Fe-rich basaltic material that dominates the martian surface (Bish et al., 2013; Ehlmann and Edwards, 2014). These four environments represent some of the diverse lithologies present on Mars, allowing for a nuanced assessment of habitability for NDFO microorganisms. These four source lithologies are not all Noachian, but the dilution used to create the media is relevant to this time period. In this study, we use these four brines to represent near-surface aqueous environments, thought to have been widespread on Noachian Mars and which may retain traces of life (Ehlmann et al., 2011).

Biosignature preservation in the martian near-surface geological record is a key issue concerning the detection of any hypothetical early martian biosphere. Microbial Fe^2+^ oxidation is responsible for numerous instances of mineralised microfossils in the terrestrial geological record (Chan et al., 2011; Chi Fru et al., 2013; Crosby et al., 2014), and NDFO specifically has been shown to promote biomineralisation of cells (Kappler et al., 2005; Miot et al., 2009a; Klueglein et al., 2014), which we have previously proposed as a potential mechanism for morphological biosignature generation on Mars (Price et al., 2018).

Potential targets for the investigation of such biosignatures on Mars could be palaeoenvironments where aqueous fluids have interacted with olivine. Olivine has been demonstrated to act as a solid Fe^2+^ source for microaerophilic Fe oxidation, enhanced by microbial weathering and dissolution of the mineral surface (Popa et al., 2012). This mineral is also a major constituent of many martian meteorites [including the Chassignites and some Shergottites (Floran et al., 1978; Nyquist et al., 2001; Goodrich, 2002; Beck et al., 2006)]. Olivine has been detected from martian orbit in close association to hydrous minerals (Ehlmann et al., 2008) and is a constituent of rocks found on the martian surface (McSween et al., 2006; McSween, 2015; Filiberto, 2017). As such, olivine is assessed here as a substrate for NDFO growth and subsequently examined for cellular structures indicating biomineralisation.

This paper is the first to explore the proposition that oligotrophic NDFO could have contributed to chemotrophic growth in near-surface fluids on early Mars, using nitrates and Fe^2+^-rich lithology as sources of electron acceptors and donors. We test the specific suitability of multiple putative early martian environments for NDFO, using a suite of media of varying compositions and an olivine bedrock analogue. In calculating the ionic compositions of these brines to match those expected for subsurface groundwaters we can apply the results of this research to habitability of both the early martian crust and potential extant subsurface reservoirs. The olivine surfaces will be inspected for signs of NDFO-driven biomineralisation behaviours that may enhance morphological biosignature preservation. In assessing the viability of NDFO in ancient martian environments and cell structure preservation on mineral surfaces, the results of this study can inform target selection for *in situ* life detection efforts, such as the Mars 2020 Perseverance rover and ExoMars Rosalind Franklin rover, and sample collection for future return. Finally, we quantify the carry-over effect in our study, examining organic carbon across blanks and media inoculated with washed and starved cells.

## Materials and Methods

### Microorganisms

Three bacterial strains, *Pseudogulbenkiania* sp. strain 2002 (DSM-18807), *Paracoccus* sp. strain KS1 (DSM-11072) and *Acidovorax* sp. strain BoFeN1 were used in this study to investigate oligotrophic growth in simulated martian fluids and separately on a Mars-relevant mineral substrate (Supplementary Figure S1). The selected strain represents three proposed categories of NDFO. *Pseudogulbenkiania* sp. strain 2002 has been reported as performing autotrophic NDFO (Weber et al., 2006b), *Acidovorax* sp. strain BoFeN1 is thought to grow mixotrophically by NDFO (Muehe et al., 2009), and *Paracoccus* sp. strain KS1 is an organotrophic denitrifier (Iordan et al., 1995). All strains were acquired from DSMZ (German Collection of Microorganisms and Cell Cultures in Leibniz, Germany) except *Acidovorax* sp. strain BoFeN1, which was obtained from the IMPMC (Institut de Minéralogie, de Physique des Matériaux et de Cosmochimie in Paris, France).

The headspace used in these experiments (90% N_2_, 10% CO_2_, 1 bar) is not intended as a facsimile of the martian atmosphere, which is now dominated by (95.9%) CO_2_ and thought to have been so in early martian history (Ramirez et al., 2014; Jakosky et al., 2017; Kurokawa et al., 2018). However, given the estimates of >0.5 bar atmospheric pressure on Noachian Mars (Kurokawa et al., 2018), the ppCO_2_ is a reasonable approximation for the scope of the experiments.

Anaerobic nutrient medium (L^−1^: 5.0 g of peptone, 3.0 g of meat extract, 5.0 g of Na_2_S_2_O_3_.5H_2_O) was used for routine growth of *Pseudogulbenkiania sp*. strain 2002, *Paracoccus sp*. strain KS1 and *Acidovorax sp*. strain BoFeN1. All cultures were incubated statically at 30°C for 48 h prior to inoculation.

### Mars Simulant-Derived Media

To investigate microbial growth in simulated martian aqueous conditions, the selected strains were grown in four Mars simulant-derived media, which were based on the geochemical composition of the following lithologies: contemporary Mars (CM), sulphur-rich (SR), haematite-rich (HR) and Shergottite (SG). The fluid ion chemistries (Supplementary Table S1) were calculated assuming complete mineral dissolution—without secondary mineral precipitation—of 1 g simulant in 1 l water, giving a water/rock ratio (W/R) of 1,000 (Ramkissoon et al., 2019). This value was chosen to represent the W/R of fluids within bedrock fractures, in a near-surface martian environment. Given the uncertainty over the longevity of open water bodies on Mars, subsurface aqueous environments were chosen as representative of a more stable martian aqueous environment.

The composition of the media (Table 1) was calculated based on the predicted ion concentrations listed in the Supplementary Data. The insoluble components listed in Table 1 (FeO, Fe_2_O_3_, 3Al_2_O_3_^.^2SiO_2_, 3MgO˙4SiO_2_^.^H_2_O, SiO_2_, TiOH, Ca(OH)_2_) were suspended in anoxic milli-Q water (1 mg L^−1^ resazurin) under N_2_ flushing, sealed and autoclaved at 121°C for 15 min. The soluble components were prepared as stock solutions in anoxic milli-Q water and filter-sterilised with a 0.2 μm filter and added to achieve the final concentrations shown in Table 1.

**Table 1:** Chemical composition of the contemporary Mars (CM), sulphur-rich (SR), haematite-rich (HR) and Shergottite (SG) media.

	Simulant			
	CM	SR	HR	SG
Component	Concentration (μM)			
NaNO_3_	1,000	618.28	746.05	–
Mg(NO_3_)_2_	–	190.86	126.98	500
Na_2_S	–	–	11.56	31.59
MnCl_2_	14.71	10.42	11.56	9.87
K_2_HPO_4_	19.83	–	72.04	78.70
NaOH	42.45	–	–	461.10
KOH	233.55	–	98.95	–
Ca(OH)_2_	1381.81	1510.99	1020.33	1692.42
FeSO_4_	226.29	2300.89	533.84	–
FeO	2392.10	578.66	1429.27	2253.63
Fe_2_O_3_	–	–	961.19	75.66
3AL_2_O_3_.2SiO_2_	329.69	164.92	233.26	211.81
3MgO.4SiO_2_.H_2_O	506.55	–	1597.01	685.33
SiO_2_	4665.92	3271.76	–	5420.55
TiOH	92.27	58.41	61.95	31.64
MnSO_4_	19.04	5.65	746.05	18.59
Na_2_HPO_4_	–	–	126.98	1.63
Fe_2_(SO_4_)_3_	220.41	210.15	11.564	–
KH_2_PO_4_	–	420.45	11.56	–
MgSO_4_	–	1131.97	72.04	–

Under anoxic conditions in a COY anaerobic chamber, 30 ml of media was dispensed into 50 ml Wheaton bottles and sealed with blue butyl stoppers. The headspaces were flushed with a 90% N_2_/10% CO_2_ headspace to remove the 5% H_2_ component of the COY anoxic atmosphere. Initial pH for these media is given in Supplementary Table S2.

Prior to inoculation, the cells were washed to remove any excess growth medium. For this, 1 ml of exponentially grown cells were harvested by centrifugation at 3000 × *g*, for 10 min. All manipulations were conducted anaerobically in the COY chamber, with sealed microcentrifuge tubes removed for the centrifugation steps. The cell pellet was washed twice with sterile simulant-derived media and resuspended to a final cell density of 10^9^ cells ml^−1^ based on cell counts at 100 × magnification. A 1% inoculum was used to inoculate the bottles, which gave an initial concentration of 10^7^ cells ml^−1^. The bottles were incubated at 25°C, without shaking, for 10 days. Each experiment was carried out in triplicate with abiotic controls prepared in parallel.

### Olivine Media Growth Cultures

To investigate growth on the surface of olivine. and potential associated biosignature formation, the strains were grown in a minimal medium using olivine as the sole source of Fe^2+^. The olivine was sourced from the Upper Loire region of France and purchased from Richard Tayler Minerals. The elemental composition and forsterite value (Fo_84_; Mg_1.68_Fe_0.32_SiO_4_) of the olivine was determined by Electron Probe Micro-Analysis (EPMA; Cameca SX 100 microprobe). Standard silicate analysis conditions were used (column conditions: 20 keV, 20 nA; beam size: 10 μm). Each culture and control contained 5 g of olivine (0.5–1 mm grain size), which had been sonicated in acetone and washed with milli-Q water to remove organics. Additionally, 1 cm^3^ polished olivine cubes, which had been acetone-washed, were added.

The olivine was added as the sole electron donor to the anoxic minimal medium (L^−1^: 0.3 g of NH_4_Cl, 0.4 g of MgCl_2_.6H_2_O, 0.1 g of CaCl_2_.2H_2_O, 0.6 g of K_2_HPO_4_, 50 mg of MgSO_4_, 30 mM NaHCO_3_, 4 mM NaNO_3_, 1 mg resazurin) after N_2_ sparging for 15 min. The NaHCO_3_ and NaNO_3_ (0.2 μm-filtered sterilised) were added after autoclaving from 1 M stock solutions. The pH was altered to 7 using 0.1 M HCL or 0.1 M NaOH and 50 ml aliquots was dispensed into 125 ml Wheaton bottles, which were sealed with blue butyl stoppers and flushed with a 90% N_2_/10% CO_2_ headspace.

The inoculum was washed and resuspended to a concentration of 10^7^ cells ml^−1^ cells, as described in the section above. A 1% inoculum was used to inoculate the medium (initial concentration of 10^5^ cells ml^−1^), and the bottles were incubated at 25°C, without shaking, for 146 days. Each experiment was carried out in triplicate with abiotic controls prepared identically in parallel.

### Organic Carry-Over

Non-purgeable organic carbon (NPOC) analysis was employed to compare washed cells with washed *and* nutrient-starved cells in order to quantify intracellular organic carbon carried into the experiment within inoculated cells.

Each strain was grown in anaerobic nutrient media for 48 h at 30°C, before twice washing, as described above, and resuspending in the test media (CM, SR, HR, SG and olivine minimal medium). The suspensions were used to inoculate anaerobic 30 ml cultures (100% N_2_ headspace) of the corresponding media, to give initial concentrations of 10^7^ cells ml^−1^, and incubated at 25°C for 48 h.

Two sets of 10 ml samples were collected, at 0 and 48 h. The samples were frozen at −20°C, heated to 90°C, and sonicated for 10 min to lyse cells. Cell debris was then removed using 0.2 μm filters. NPOC was quantified in lysed samples using a Shimadzu TOC-L CPH analyser with associated ASI-V auto-sampler (detection limit = 1 mg L^−1^) by the British Geological Survey (Keyworth, UK; Marriott et al., 2020). The analysis used a Peak Scientific 42-1,040 TOC gas generator for carrier gas of high purity air, with TOC Control V Software (version 1.09) package was used to control the analyser and acquire data. Extracted fluid (<6 ml) was pipetted into glass tubes, capped by aluminium foil and placed into the sample carousel for analysis. A Certified Reference Material (CRM 100 mg L^−1^ Carbon from National Institute of Standards and Technology—ERA®) was used (10 mg L^−1^) of C during each analytical run to test performance. Sample and calibration standards were run together, and the results accepted with a coefficient of variation (CV) <5% at concentrations greater than three times the quantification limit, or <20% at concentrations less than three times the quantification limit. The analytical error was estimated at 8%.

### Monitoring Microbial Growth

To monitor microbial growth in the four Mars simulant-derived media, protein biomass was measured using a modified Bradford assay (Miot et al., 2009a). Tamm reagent was used to dissolve the Fe^3+^ oxides in the media to overcome suspected interference of metal oxides with the action of the Bradford reagent and cell stains. A secondary function of Tamm reagent is cell lysis (Vodyanitskii, 2001), meaning protein quantification was preferred to cell counts in the simulant-derived media cultures. Cell counts were taken from 100 μl samples in the olivine cultures using a Baclight cell viability kit (Invitrogen) and analysed using a Leica DMRB microscope equipped with epifluorescence (Leica Microsystem, Bensheim, Germany). The growth rate constant (*k*) for the log phase of growth was determined by plotting the natural log of the protein concentration and cell counts over time (Pirt, 1975).

### Chemical Analysis

To monitor the concentration of Fe^2+^ and total Fe throughout the Mars simulant-derived media experiments, the Ferrozine method was used. At each time point, two 20 μl samples from each culture and control were collected anaerobically and diluted *via* a 0.2 μm filter into 980 μl of a 0.5 M HCl solution and 980 μl of a reducing solution (0.5 M HCl, 0.3 M Hydroxylamine hydrochloride), respectively, and incubated at 4°C for 1 h. The HCl solution prevents abiotic oxidation of Fe^2+^ in the sample, allowing determination of Fe^2+^ concentration. The reducing solution converts all Fe^3+^ in the sample to Fe^2+^, which can then be measured to give a total Fe (Fe_total_) concentration from which Fe^2+^/Fe^3+^ can be deduced. A 20 μl aliquot of each digested sample was mixed with 980 μl of Ferrozine solution in a cuvette and absorbance measured at 562 nm on a spectrophotometer. Sample concentrations were calculated from a standard curve at 10 mM, 5 mM, 1 mM and 500 μM FeSO_4_.

Inductively coupled plasma mass spectrometry (ICP-MS) was used to measure the initial and final Fe concentrations during the olivine experiment. Ferrozine could not be used because of the low concentrations of dissolved Fe in solution. An aliquot (9 ml) was extracted under anaerobic conditions using a N2-flushed sterile syringe at day 146. The sample was filtered through a 0.2 μm sterile filter into 1 ml aliquots of 20% HNO_3_ solution, resulting in 2% final HNO3 concentration. ICP-MS was conducted using an Agilent 7,500 s with New Wave 213 laser system at The Open University, United Kingdom. Detection limits of the instrument are listed in Supplementary Table S3.

The nitrite concentration was measured using the Griess reagent assay, in which sulfanilic acid reacts with 1-naphthylamine to produce red-pink azo compounds in the presence of nitrite ions (Griess, 1879). For monitoring nitrite concentrations, a 100 μl aliquot of culture was transferred into sterile 1.5 ml microcentrifuge tubes and centrifuged at 15,500 × *g* for 10 min. In parallel, nitrite standards were prepared by diluting 100 mM NaNO_2_ with 0.1 M NaOH solution, to give 100 μM, 50 μM, 25 μM, 10 μM, 5 μM, 2.5 μM and 1 μM NaNO_2_ concentrations. The standards (in triplicate) and the culture supernatants (50 μl) were transferred to a 96-well flat-bottomed, optically clear ELISA microplate. 100 μl of 1 × Griess reagent solution (Sigma-Aldrich) was added to each well. After 15 min, the absorbance was read using a Bio-tek ELx808 microplate reader with a 540 nm filter, using KC4 software for the data output.

The nitrate concentration was measured using an ELIT 0821 ion selective NO_3_^−^ electrode with ELIT 003 lithium acetate reference electrode (Nico2000) connected to a conductivity meter (HANNA instruments), as per the manufacturer’s instructions. The electrode was calibrated by placing the electrode in 5 ml of electrode buffer solution and waiting for equilibrium to be reached.

pH was measured using a Thermo Scientific Orion Three Star pH meter with a two-point calibration using Omega Buffer solutions at pH 4 and 7 at the start and end points of the experiment.

### Morphological Analyses

To investigate morphological biosignatures, the olivine cubes were removed from the culture after 146 days. After drying in covered, sterile glass vials under a 85% N_2_/10% CO_2_/5% H_2_ atmosphere for 2 days, the rocks were gold-coated under vacuum. Analysis was carried out under vacuum with a Zeiss Supra 55 VP Field Emission Gun Scanning Electron Microscope (FEG-SEM), using SE2 and Cent detectors at ´100 to ´50,000 magnifications. For imaging, working distances of 4.1–10.1 mm and accelerating voltages of 3–20 kV were used. Energy-dispersive X-ray spectroscopic (EDS) analysis was performed using the integrated Aztec Energy v3.3 system (Oxford Instruments).

### Statistics

Significant differences between the cultures and controls were tested using a 2-tailed paired Student’s t-test. Pooled biotic samples were tested for significance against controls using a 2-tailed 2-sample t-test assuming equal variance. Correlations were calculated using Pearson’s coefficient of linear correlation.

## Results

### Mars Simulant-Derived Media Experiment

#### Initial Conditions

The initial pH of the four simulant-derived media was similar (6.69–7.00), with sulphur-rich (SR) the most acidic and Shergottite (SG) the most alkaline, as shown in Supplementary Table S2. Initial nitrate ion concentration was equalised across the four media at 1 mM, whereas dissolved Fe^2+^ ranged from ~2.3 mM in SR to below detection limits in SG (where Fe was present as insoluble FeO; Table 1).

#### Growth In Martian Simulant-Derived Media

**Figure 1:**
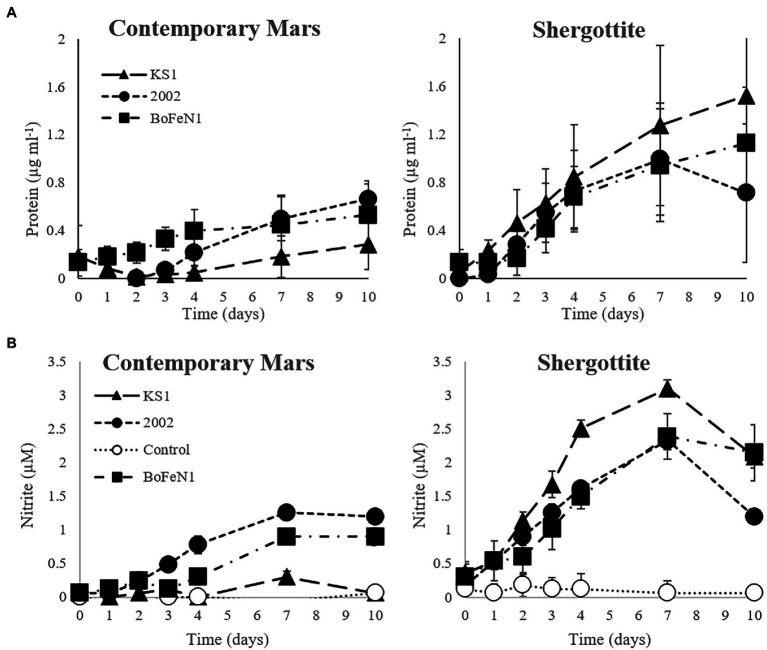
**(A)** Protein and **(B)** nitrite concentration over time in the contemporary Mars (CM) and Shergottite (SG) simulant media for *Paracoccus* sp. strain KS1, *Pseudogulbenkiania* sp. strain 2002, *Acidovorax* sp. strain BoFeN1 (± standard error of triplicates). Protein values are normalised against the abiotic control.

The Shergottite (SG) and contemporary Mars (CM) media were able to support growth (Figure 1A), whereas no significant growth was observed for any organism in the haematite-rich (HR) or sulphur-rich (SR) media (Supplementary Figure S2).

For the SG medium, the mean specific growth rates (*k*) for the triplicates were 0.25, 0.15, and 0.10 day^−1^ for *Pseudogulbenkiania* sp. strain 2002, *Paracoccus* sp. strain KS1 and *Acidovorax* sp. strain BoFeN1, respectively. In the CM medium, *k* was similar for *Pseudogulbenkiania* sp. strain 2002 (0.14 day^−1^) and *Acidovorax* sp. strain BoFeN1 (0.13 day^−1^). No significant protein concentrations were detected over 10 days in normalised data for *Paracoccus* sp. strain KS1 in CM, thus growth of *Paracoccus sp*. Strain KS1 cannot be confirmed. Significant differences between growth in SG and CM media were not established for any strain.

#### Nitrite Production and Nitrate Reduction

There was an overall positive correlation between nitrite and protein concentrations in both the CM and SG media (Supplementary Figure S3). Furthermore, strains that produced a significant increase in protein concentration in the CM (*Pseudogulbenkiania* sp. strain 2002 and *Acidovorax* sp. strain BoFeN1) and SG (all strains) media, demonstrated positive correlations between nitrite concentration (Figure 1B) and protein, detailed in Supplementary Table S4. Negative correlations were observed both between the nitrate and nitrite concentrations (*ρ* = −0.87 at day 10) and between nitrate and protein (*ρ* = −0.89 at day 10) across the same strains in CM and SG media (Supplementary Figure S4), indicating more extensive consumption of nitrate in growing cultures. The maximum protein and nitrite concentrations were observed in the *Paracoccus* sp. strain KS1 SG cultures. The maximum nitrite concentrations (at day 7) were significantly greater (*p* < 0.05) for each strain in the SG medium compared to the values in the corresponding CM medium. There was no significant production of nitrite or nitrate consumption in the abiotic controls for the simulant-derived media (*p* < 0.05).

#### Fe Oxidation State

Over the duration of the experiment, the Fe in the CM media became reduced in both the biotic and abiotic controls (Supplementary Figure S5). The mean Fe^2+^/Fe_total_ ratios (shown in Table 2) were lower in the three CM biotic culture sets than in the control and there were inverse correlations between Fe^2+^/Fe_total_ relative to the control and microbial growth in the CM media for *Paracoccus* sp. strain KS1 (*ρ* = −0.43), *Pseudogulbenkiania* sp. strain 2002 (*ρ* = −0.45) and *Acidovorax* sp. strain BoFeN1 (*ρ* = −0.92; Supplementary Data). However, these trends did not result in significant differences in Fe^2+^/Fe_total_ ratios between the CM cultures and controls within the timeframe of the experiment, either when cultures were considered in organism-specific groups (*Acidovorax* sp. strain BoFeN1 *p* = 0.22, *Pseudogulbenkiania* sp. strain 2002 *p* = 0.36, *Paracoccus* sp. strain KS1 *p* = 0.06) or a pooled biotic sample set (*p* = 0.09). Macroscopic Fe oxide precipitates were not observed in any of the cultures. Dissolved Fe was not detected in SG media by the Ferrozine assay, as the Fe sources used were insoluble.

**Table 2:** Change in dissolved Fe, nitrate, Fe^2+^/Fe_total_ ratios and pH for Mars simulant-derived media cultures and abiotic controls over 10 days (± standard error of triplicates), growth rate (*k*) and doubling time of growing cultures.

Media	Inoculum	∆NO_3_^−^	∆Fe^2+^/Fe_total_	End point pH	∆pH	*k* (day^−1^)	Doubling time (days)
Contemporary Mars	*Paracoccus* sp. strain KS1	−2.32% (±4.49)	+0.163 (±0.056)	6.28 (±0.06)	−0.47 (±0.06)	0.06	5.10
	*Pseudogulbenkiania* sp. strain 2002	−14.17% (±2.93)	+0.171 (±0.026)	6.39 (±0.07)	−0.36 (±0.07)	0.14	2.11
	*Acidovorax* sp. strain BoFeN1	−9.95% (±3.07)	+0.178 (±0.048)	6.21 (±0.03)	−0.54 (±0.03)	0.13	2.26
	Control	−2.61% (±1.28)	+0.266 (±0.061)	6.28 (±0.07)	−0.47 (±0.07)	−	−
Sulphur-rich	*Paracoccus* sp. strain KS1	−2.46% (±3.40)	−0.090 (±0.154)	5.85 (±0.02)	−0.84 (±0.02)	−	−
	*Pseudogulbenkiania* sp. strain 2002	−3.96% (±3.67)	−0.130 (±0.093)	5.86 (±0.02)	−0.83 (±0.02)	−	−
	*Acidovorax* sp. strain BoFeN1	−3.96% (±3.67)	−0.140 (±0.150)	5.76 (±0.05)	−0.93 (±0.05)	−	−
	Control	−2.61% (±1.28)	−0.062 (±0.161)	5.79 (±0.01)	−0.90 (±0.01)	−	−
Haematite-rich	*Paracoccus* sp. strain KS1	−5.67% (±1.22)	+0.051 (±0.055)	6.36 (±0.03)	−0.36 (±0.03)	−	−
	*Pseudogulbenkiania* sp. strain 2002	−2.54% (±2.50)	−0.122 (±0.056)	6.34 (±0.02)	−0.38 (±0.02)	−	−
	*Acidovorax* sp. strain BoFeN1	−2.61% (±1.28)	−0.027 (±0.113)	6.30 (±0.04)	−0.42 (±0.04)	−	−
	Control	−0.96% (±2.63)	−0.066 (±0.072)	6.31 (±0.02)	−0.41 (±0.02)	−	−
Shergottite	*Paracoccus* sp. strain KS1	−20.85% (±1.79)	−	6.70 (±0.01)	−0.30 (±0.01)	0.15	2.02
	*Pseudogulbenkiania* sp. strain 2002	−11.45% (±2.27)	−	6.74 (±0.06)	−0.26 (±0.06)	0.25	1.22
	*Acidovorax* sp. strain BoFeN1	−9.75% (±4.55)	−	6.63 (±0.03)	−0.37 (±0.03)	0.10	2.91
	Control	−1.04% (±1.28)	−	6.67 (±0.07)	−0.33 (±0.07)	−	−

#### pH

Overall, the pH decreased and there were no significant differences between the biotic and abiotic experiments after 10 days (*p* > 0.05), except for *Pseudogulbenkiania* sp. strain 2002 grown in the SG medium where pH decreased less than in the abiotic control (*p* = 0.02). Values are shown in Table 2.

### Olivine Culture Experiment

#### Microbial Growth With Olivine as a Sole Source of Fe

**Figure 2:**
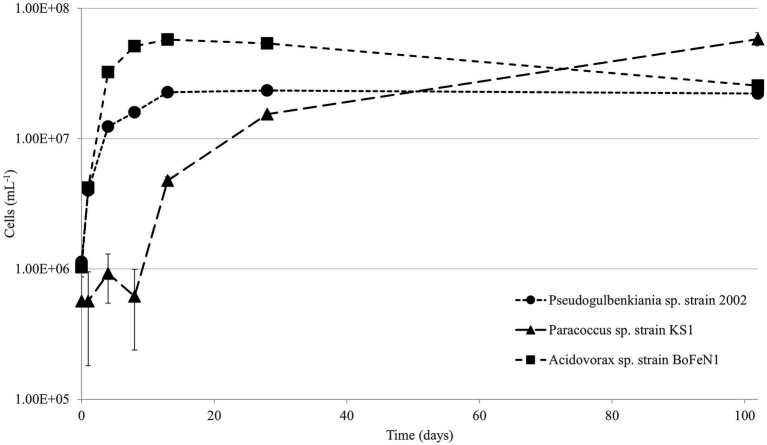
Viable cell counts over time for *Pseudogulbenkiania* sp. strain 2002, *Paracoccus* sp. strain KS1 and *Acidovorax* sp. strain BoFeN1. Error bars represent ± standard error.

Each of the strains was able to grow in the minimal medium using olivine as a sole source of Fe^2+^ (Figure 2). The specific growth rates varied between 0.07 and 0.37, as shown in Table 3. There was a negative correlation (*ρ* = −0.67) between maximum cell numbers and nitrate concentration at the end of the experiment (Supplementary Figure S6), meaning growth is positively correlated to nitrate consumption.

**Table 3:** Final Fe concentrations, changes in nitrate and pH for inoculated olivine culture series and abiotic controls (± standard error of triplicates), growth rate (*k*) and doubling time for growing cultures.

Series	∆Fe_total_ (μM)	∆NO_3_^−^	∆pH	*k* (day^−1^)	Doubling time (days)
*Paracoccus* sp. strain KS1	+0.182 (±0.029)	−40.28% (±3.79)	+0.12 (±0.00)	0.07	4.31
*Pseudogulbenkiania* sp. strain 2002	+0.154 (±0.025)	−34.80% (±3.07)	+0.15 (±0.04)	0.26	1.16
*Acidovorax* sp. strain BoFeN1	+0.140 (±0.027)	−27.93% (±0.76)	+0.14 (±0.02)	0.37	0.80
Control	+1.000 (±0.017)	−10.20% (±0.95)	+0.13 (±0.01)	–	–

ICP-MS analysis after 146 days (Table 3) demonstrated that the concentration of Fe was significantly lower in the aqueous media of the biotic experiments compared to that of the abiotic control (*p* < 0.05), indicating that Fe is being removed from solution. However, no macroscopic Fe oxide precipitates were observed.

The pH of the cultures and abiotic controls increased significantly over the course of the experiment (*p* < 0.05), as shown in Table 3. However, there were no significant differences in the end point pH between any of the cultures and the abiotic control (*p* > 0.05).

#### Morphological Biosignatures

**Figure 3:**
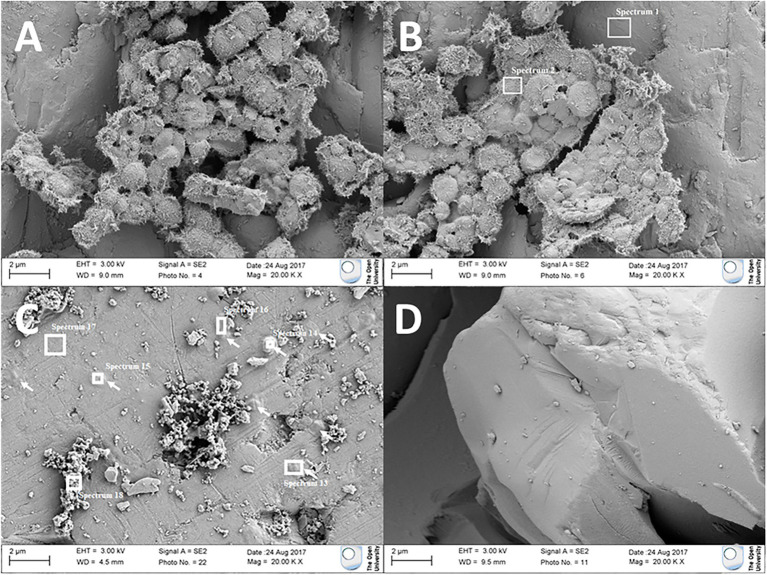
Electron micrographs of **(A,B)** coccoid features adhered to olivine after culture with Pseudogulbenkiania sp. strain 2002. **(C)** Flattened, rounded features (indicated by arrows) adhered to the olivine surface after culture with *Paracoccus* sp. strain KS1. Target areas for electron dispersive X-ray spectroscopic (EDS) analysis are shown with white boxes. No such features were observed in **(D)** the abiotic control.

FEG-SEM analysis was performed on olivine cubes after 146 days of incubation. Cell-like features were observed on the mineral surface in *Pseudogulbenkiania* sp. strain 2002 and *Paracoccus* sp. strain KS1 cultures. For *Pseudogulbenkiania* sp. strain 2002, the individual features measured approximately 1.5–2 μm in diameter. The clusters typically composed of between 50 and 100 coccoid units, which were covered by a layer of nanometre-scale dendritic features (Figure 3). EDS analysis demonstrated a co-location between the coccoid features and regions with elevated C (11.7 ± 0.4 wt%) when compared with the underlying substrate (4.0 ± 0.3 wt%; Figure 3B).

Individual coccobacillal (ovoid) structures, flatter than those observed in the *Pseudogulbenkiania* sp. strain 2002 cultures and ~ 1 μm in diameter, were also observed attached to the mineral surface of the *Paracoccus* sp. strain KS1 olivine cube. The thickest of these features were also co-located with areas of elevated carbon (6.0 ± 0.4 wt%) relative to the substrate (1.8 ± 0.3 wt%; Figure 3C). No features suggestive of biological processes were observed on the olivine mineral surfaces in the abiotic control.

### Residual and Carried Over Organic Carbon

No significant differences (*p* = <0.05 in all cases) in NPOC were found between starved and non-starved inocula. This was true across the five media used and for each of the organisms, as well as in abiotic controls. The mean normalised NPOC concentrations are given in Supplementary Table S5, with significance values. There was a detectable baseline of residual organic carbon in the five blank media ranging from 2.23 mg L^−1^ in CM to 4.51 mg L^−1^ in SG.

## Discussion

### Nitrate Reduction Drives Growth in Simulated Martian Environments

The presence of nitrates in modern and ancient martian surface samples (Stern et al., 2015) greatly diversified the range of metabolisms hypothetically possible, bringing autotrophic and heterotrophic forms of nitrate reduction and denitrification to the fore.

The abundances of nitrate on Mars in scooped dust and drilled sediment are in the range of 70–1,100 ppm (Stern et al., 2015), equivalent to ~1–16 mM and sufficient to drive nitrogen-based metabolisms in the presence of potential active nitrate formation mechanisms, such as volcanic lightning, impact-generated and radiation-catalysed nitrogen fixation reactions (Mahaffy et al., 2013; Smith et al., 2014; Stern et al., 2015). Denitrifying microbes are typically active in environments with lower nitrate concentrations (<1 ppm), but occur across a wide range of concentrations and have been described performing denitrification at up to 36,000 ppm (0.58 M) on Earth, albeit at an impaired rate (Glass and Silverstein, 1999; Tomasek et al., 2017).

The growth of *Acidovorax* sp. strain BoFeN1, *Pseudogulbenkiania* sp. strain 2002 and *Paracoccus* sp. strain KS1 with olivine and in the SG simulant media, and the two former strains in the CM simulant media, suggests that growth by nitrate reduction is possible on both martian mineral substrates and in simulated martian fluid chemistries. This is relevant for early biospheres on Mars, given the availability of nitrate as an electron acceptor within the context of the early surface environment (Stern et al., 2015), and the near absence of molecular oxygen.

### Oligotrophic Mixotrophy Over Autotrophy

Of the microorganisms investigated here, autotrophic growth by NDFO has only been reported in *Pseudogulbenkiania* sp. strain 2002, which was claimed to have grown consistently across repeated transfers (Weber et al., 2006b). In comparison with the olivine cultures in this study, the autotrophic growth observed by Weber et al. (2006b) occurred with a higher growth rate. However, there was no limitation of Fe^2+^ in that previous study, which invoked an initial concentration of 10 mM. Under the Fe^2+^-limited conditions of this experiment (only solid-state Fe and <1 μM Fe in solution), it is unsurprising that we observed a lower growth rate here.

Although *Acidovorax* sp. strain BoFeN1 and *Paracoccus* sp. strain KS1 had previously been thought to require an organic co-substrates for NDFO-associated growth (Iordan et al., 1995; Kappler et al., 2005), both strains grew on olivine and in SG cultures with no organic media component. The reason for this is likely the low-but-detectable baseline concentration of organic carbon which was detected in all of the blank media, identified in this case as originating from the media components. The NPOC concentrations detected (~2.2–4.5 mg L^−1^) were equivalent to the presence of ~12–25 μM glucose, meaning these compounds could be serving as a co-electron donor for growth by mixotrophic NDFO in otherwise autotrophic cultures. This conclusion supports the assertion by Bryce et al. (2018) that true autotrophic growth by NDFO has only been conclusively demonstrated in one instance—the so-called ‘KS’ co-culture (Straub et al., 1996; Blöthe and Roden, 2009) – and that most reported NDFO strains are in fact mixotrophic or organotrophic denitrifiers. Even so, the growth of strains as metabolically and phylogenetically diverse as *Acidovorax* sp. strain BoFeN1, *Pseudogulbenkiania* sp. strain 2002 and *Paracoccus* sp. strain KS1 under low Fe^2+^ availability and oligotrophic, anoxic conditions supports the suitability of NDFO organisms to circumneutral early martian environments, which are now thought to have been replete with organic carbon compounds (Eigenbrode et al., 2018).

### Diverse Martian Chemistries Affect NDFO Habitability

**Figure 4:**
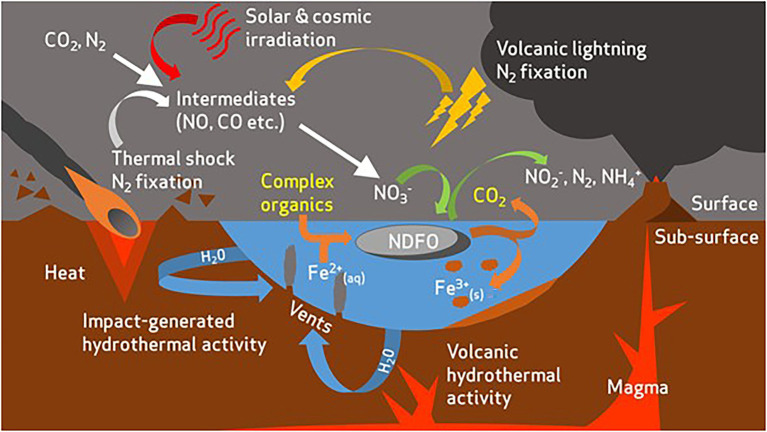
Overview of potential redox substrate sources for nitrate-dependent Fe^2+^-oxidising microorganisms in the early Mars environment [modified from Price et al., 2018]. Complex organics are present as an additional electron donor and carbon source for microbial life. Organics act as a co-substrate with Fe^2+^ to drive respiratory nitrate reduction and ATP production in NDFO-performing microorganisms. CO_2_ is produced as a metabolite from oxidation of organics.

The Shergottite meteorites, upon which the SG medium is based (Ramkissoon et al., 2019), are proposed to be representative of martian basaltic terrains (Treiman, 2003). With that in mind, the growth of all three strains in the SG medium indicates that the geochemical composition of basaltic environments could support the proliferation of NDFO microbes if sufficient nitrate was present as a result of various formation mechanisms (Stern et al., 2015, 2017; Figure 4). This finding has implications for life at both the early surface of Mars, and in the subsurface, where basalts of this type have been abundant throughout the planet’s history (Bertka and Fei, 1998; Bridges and Warren, 2006; Steele et al., 2012). Compared to the other simulant-derived media, the SG medium is particularly characterised by the presence of insoluble Fe components. Paired with the growth of all three strains on an olivine substrate, this finding suggests that oligotrophic NDFO may perform optimally under Fe^2+^ limitation, possibly due to additional ATP consumption for metal efflux under high Fe^2+^ conditions.

The CM medium contains greater concentrations of dissolved Fe^2+^, Fe^3+^ and sulphate in comparison to SG medium. It is derived from a simulant of aeolian dust at Gale crater and better represents the modern global composition of the weathered martian surface (Ramkissoon et al., 2019), so, the confirmed growth of *Pseudogulbenkiania* sp. strain 2002 and *Acidovorax sp*. strain BoFeN1 (evidence for growth of *Paracoccus* sp. strain KS1 was inconclusive) could mean that near-surface aqueous environments with regolith of a similar composition could support NDFO organisms. Habitation of hypothetical modern and ancient fluids by NDFO microbes is made more plausible by the evidence for Amazonian hydrological activity (Adeli et al., 2016; Butcher et al., 2017). Butcher et al. (2017) described eskers, subglacial landforms caused by basal melting and subsequent erosion, revealed by retreating glaciers in areas of elevated geothermal heat flux. The existence of these features indicates that Mars has hosted large-scale mixing of meltwater with modern surface geology, potentially providing environments similar to the CM media in this experiment. If a putative early biosphere existed and retreated to the deep subsurface, as others have suggested (Michalski et al., 2013), localised geothermal activity paired with glacial basal melting could have provided an opportunity to periodically replenish the near-surface environments with extant microbes and generate more recent biosignatures.

The HR and SR media, in which no strain grew, hold some key differences in initial chemistry and progression of physicochemical conditions throughout the experiment, which may help to explain the observed variation in habitability. The HR media contained a higher dissolved concentration of Fe^2+^ than either SG or CM. Counterintuitively, this may have had an inhibitory effect on the strains tested. Carlson et al. (2012) describe the toxic reactions of Fe^2+^ with oxidised nitrogen species, which may have inhibited growth if the cultures were not able to acclimatise following inoculation. Alternatively, the cells may have undergone rapid encrustation leading to loss of viability, as described previously for *Acidovorax* sp. strain BoFeN1 (Miot et al., 2009a).

The concentration of sulphates (>3.6 mM) in SR media greatly exceeded either CM (0.47 mM) or SG (0.05 mM) and caused acidification. For example, after 10 days the pH had dropped below the reported range (pH < 6) for NDFO metabolism. Acidification reduces the energetic favourability of the redox potential NDFO couple for sustaining microbial growth and carbon fixation (Weber et al., 2006a). The acidification could also be exacerbated by the reaction of the 10% CO_2_ headspace component with water to form carbonic acid and the lack of buffer capacity in solution, as the other three simulant media also became acidified (albeit to a lesser extent than SR; Supplementary Table S2). The martian atmosphere is, and has been, primarily composed of CO_2_ throughout history, so the acidifying effects of this on long-lived hydrological systems in contact with the atmosphere are important to note when considering habitability for pH-dependent metabolisms, such as NDFO. Indeed, acidified oceans (pH < 6.2) have been proposed on early Mars as a consequence of a CO_2_ (0.8–4 bar) atmosphere (Fairén et al., 2004). If true, these conditions would inhibit NDFO as a viable metabolism across surface-exposed water bodies of early Mars. However, basaltic rocks (relevant to the olivine and SG cultures) act as a buffer and systems are generally neutral to alkaline when not in direct contact with the atmosphere or any other source of acidification (Zolotov and Mironenko, 2007; McAdam et al., 2008). Direct geochemical analysis of rocks *in situ* at Gale crater (upon which CM is based) also show that circumneutral systems did persist on early Mars (Grotzinger et al., 2014), particularly in the subsurface, where they could even exist today (Michalski et al., 2013).

### Fe Oxidation State

Despite evidence for both microbial growth and microbially mediated nitrate to nitrite reduction in the SG and CM simulant media, there was no significant oxidation of Fe^2+^. This contrasts with expectations for active NDFO-based energy metabolisms in growing cultures (Miot et al., 2014a). For example, Weber et al. (2006b) observed that *Pseudogulbenkiania* sp. strain 2002 oxidised ~2 mM Fe^2+^ of a medium containing 10 mM Fe^2+^ over 7 days during autotrophic growth. *Acidovorax* sp. strain BoFeN1 oxidised ~2 mM of 4 mM total Fe^2+^ during autotrophic culture over 20 days (Kappler et al., 2005). These studies included higher concentrations of both Fe^2+^ and nitrate than any of the media tested here, which may account for some of the difference in the extent of Fe^2+^ oxidation when compared to the CM and SG martian simulant-derived media. It is also possible the growth observed here in CM (>1 μg ml^−1^ protein) may be insufficient to generate significant Fe oxidation relative to the control, given the sensitivity of the ferrozine assay. In comparison, Miot et al. (2009a) recorded ~65 μg ml^−1^ protein generated during mixotrophic oxidation of 5.4 mM Fe^2+^ by *Acidovorax* sp. strain BoFeN1.

There were no significant differences in Fe oxidation state between the inoculated cultures and controls for any of the simulant-derived media, which suggests that the reducing trend in the CM Fe^2+^/Fe_total_ ratios over time were predominantly the result of an abiotic chemical factor. It is noteworthy that in all of the CM cultures which showed evidence of microbial growth, the mean Fe oxidation state was more oxidised (Fe^2+^/Fe_total_ = 0.41–0.43) after 10 days than in the CM control (Fe^2+^/Fe_total_ = 0.48). Furthermore, there was a negative correlation between microbial growth and the Fe^2+^/Fe_total_ ratio—greater growth leads to greater Fe oxidation—relative to the control in CM media. That correlation suggests that growth had an oxidising effect on the media and sufficient microbial growth by nitrate reduction to nitrite in this media could produce significant patterns of Fe oxidation. However, observing this effect definitively may require a longer experiment duration, a more sensitive method for monitoring Fe oxidation state or a defined organic co-substrate to increase microbial activity.

### Oligotrophic Growth on Olivine

All three strains were able to grow using olivine as a sole source of Fe^2+^. The end point concentration of dissolved Fe was highest in the abiotic control, despite Fe being absent from the initial media composition. It can be assumed that this represents Fe (namely, Fe^2+^) leached abiotically from the olivine substrate over the duration of the experiment, which is in line with olivine dissolution kinetics at circumneutral pH proposed by Wogelius and Walther (1991) and represents a rate-limiting factor for microbial Fe^2+^ oxidation. The Fe concentrations accumulated from olivine dissolution over the course of the experiment reached a maximum of ~1 μM. In comparison, the initial Fe^2+^ concentration was ~226 μM in the CM media, whereas dissolved Fe^2+^ was below the limit of detection in SG. A similar experiment demonstrated that neutrophilic growth by microaerophilic bacteria could occur under microoxic (1.6% O_2_) conditions using olivine sand as a sole Fe source (although Fo_91_ compared with Fo_84_ in this study; Popa et al., 2012). That isolate, *Pseudomonas* sp. strain HerB, was able to grow with the same ratio of olivine mass to media volume and the same temperature and pH range as in the experiments described in this study. This adds credence to the conclusion that the rate of dissolution and concentration of Fe seen in the results presented here are indeed sufficient to help drive microbial growth. Furthermore, the anaerobic conditions and use of nitrate as an electron acceptor in the experiments detailed here may represent early martian environments more closely as they are now better understood.

Soluble Fe^2+^ concentrations were lower than the control in the actively growing cultures of *Acidovorax* sp. strain BoFeN1, *Pseudogulbenkiania* sp. strain 2002 and *Paracoccus* sp. strain KS1. Although formation of macroscopic Fe^3+^ precipitates was not observed, the most likely explanation in the context of this experiment is that micromolar Fe^2+^ is being scavenged and oxidised during NDFO, resulting in the precipitation of microscopic insoluble Fe^3+^ compounds, as has been described extensively in the literature (Schädler et al., 2009; Miot et al., 2009a, 2014a, 2015; Pantke et al., 2012). The consumption of Fe^2+^ and nitrate, together with evidence of growth from cell counts, suggests that microbial growth facilitated by NDFO has occurred. Growth of *Acidovorax* sp. strain BoFeN1 using Fe^2+^ from olivine (in which soluble Fe^2+^ was detectably consumed) was significantly greater than when grown in the SG and CM media (in which microbial Fe^2+^ consumption was not detectable). This disparity is likely to be due to the higher initial nitrate concentration in the olivine cultures (4 mM), compared to the simulant-derived media (1 mM).

### Biogenicity and NDFO Detection on Mars

SEM analysis of the olivine mineral surfaces showed features with aspects suggesting biogenic origin, some of which were supported by results from EDS analyses. The clustered features observed with the *Pseudogulbenkiania* sp. strain 2002 (Figure 3) were morphologically similar to that of *Pseudogulbenkiania* sp. strain 2002 cells, which have been visualised previously (Zhao et al., 2013). The largest clusters of mineralised cells (*Pseudogulbenkiania* sp. strain 2002; Supplementary Figure S7) retained detectable, elevated carbon signatures. These biomineralised microbial structures demonstrate a mechanism for morphological biosignature production from oligotrophic NDFO cultures. As in the *Pseudogulbenkiania* sp. strain 2002 culture, the carbon signature (Supplementary Figure S8) detected in the *Paracoccus* sp. strain KS1 culture (Figure 3C) provides supporting evidence for the biogenicity of these features. Furthermore, transmission electron microscopy has previously demonstrated the nucleation of ferric minerals in the periplasm of *Acidovorax* sp. strain BoFeN1 (Kappler et al., 2005; Schädler et al., 2009; Miot et al., 2009a, 2014a, 2015; Pantke et al., 2012; Zhou et al., 2016).

The difficulty in terms of *in situ* detection on Mars, however, is the scale of these structures (~1–200 μm). For reference, the spot sizes on the Raman Laser Spectrometer (Rosalind Franklin rover) and Scanning Habitable Environments With Raman & Luminescence for Organics & Chemicals (SHERLOC) instrument (Perseverance rover) are both 50 μm (Beegle et al., 2015; Rull et al., 2017). The consequence of this is that small biomineralised structures would be difficult to identify, even if located in the path of the beam. Even so, the features observed in this study suggest that olivine-rich rocks with historical exposure to circumneutral fluids are a logical starting point for these biosignature-oriented missions, which aim to investigate preserved Noachian sedimentary environments at Oxia Planum and Jezero crater (3.8–3.9 Ga), respectively (Goudge et al., 2018; Quantin-Nataf et al., 2019). A logical progression from the results of this paper would be to investigate NDFO encrustations using ground-based Raman instruments, in order to better interpret future rover data.

Our knowledge of the long-term effects of geological processing on NDFO-mediated cell encrustations is limited. However, Glasauer et al. (2013) combined laboratory encrustation experiments using a NDFO isolate with field observations to highlight the importance of the redox environment in fossilisation, finding that reductive dissolution could inhibit Fe biomineralisation; in testing the diagenetic maturation of organo-ferric structures from microaerobic Fe oxidisers, Picard et al. (2015) found that Fe^3+^ oxides could enhance structural and chemical preservation of biological material under high temperature and pressure and that spectroscopy could be used to identify these biosignatures in the rock record. Meanwhile, other Fe encrustations have been proposed as some of the earliest evidence for life on Earth (Li et al., 2013). Further artificial maturation experiments and identification of NDFO structures in terrestrial samples should be pursued to better understand how these biosignatures may present in target rocks on Mars.

The growth of strains in the basaltic SG medium supports this assessment, while growth in CM medium validates the hypothesis that circumneutral, sedimentary palaeoenvironments on Mars are ideal targets for NDFO biosignature detection. Likewise, the lack of microbial metabolism or growth among the tested strains in HR and SR media can be useful in guiding target selection. The acidifying, high-sulphate conditions in SR cultures are not conducive to circumneutral NDFO activity, meaning acidic sulphur-rich martian palaeoenvironments are poor candidates to host biomineralised NDFO cells. However, previous work has established the possibility of other metabolisms which may have been suited to such locations (Amils et al., 2014; Bauermeister et al., 2014). Similarly, the oxidised conditions of Fe^3+^-rich HR media may be unsuitable for NDFO organisms and suggests haematite-dominated martian lithologies would not represent good targets for NDFO biosignature detection. However, as Fe^2+^ oxidisers, these microbes are capable of producing a broad range of Fe^3+^ oxides under reducing conditions (Kappler et al., 2005; Schädler et al., 2009; Miot et al., 2009b, 2014a,b; Pantke et al., 2012). Therefore, oxidised features in otherwise reduced habitable palaeoenvironments would represent logical targets for analysis and sample caching. For identification of structures on the scale of those observed in this study, Mars sample return is vital.

The evident difficulty in determining biogenicity from morphology, even in batch cultures where the original morphology of the cells is known, serves to highlight a necessity for multiple lines of evidence when investigating potential biosignatures on Earth and Mars. Indeed, some have suggested that many putative microfossils in the rock record on Earth may require reclassification (McMahon et al., 2021). The verification of biogenicity based on morphology has led to many contentious claims about evidence of life in both ancient terrestrial rocks and martian meteorites (McKay et al., 1996; Mojzsis et al., 1996). The results of this experiment suggest that pairing morphology and elemental observations with alternative methods of determining the past presence of biological Fe metabolism, such as isotope fractionation patterns (Anand et al., 2006), should be investigated.

## Conclusion

This is the first study to experimentally investigate nitrate-dependent Fe^2+^ oxidation as a plausible metabolism for early Mars, monitoring microbial growth and NDFO under simulated martian chemical conditions and on Mars-relevant olivine mineral surfaces.

All three tested strains were confirmed to have grown in the SG media and at least two strains in the CM media, based on protein, nitrite and nitrate data, which suggests some aqueous environments on early Mars were potentially habitable for NDFO microorganisms. However, there was no significant Fe oxidation or removal of soluble Fe from solution to form Fe^3+^ oxide precipitates by cultures in any of the simulant-derived media relative to their abiotic controls, suggesting that NDFO by actively growing microbes under these conditions did not dominate the overall redox chemistry of the media.

All three strains were also able to grow under anoxic, oligotrophic conditions by respiring nitrate and utilising Fe^2+^ released by dissolution from an olivine substrate. This capability holds implications for the habitability of early Mars, where comparable conditions are likely to have existed. Signs of biomineralisation were observed on some of the olivine surfaces, demonstrating that NDFO could be a mechanism for preservation of morphological biosignatures from the early martian environment for detection in the present day. However, the small size of biomineralised features complicates *in situ* detection and necessitates Mars sample return.

## Data Availability Statement

The raw data supporting the conclusions of this article will be made available by the authors, without undue reservation.

## Author Contributions

AP conducted all experimental work in this study, with exceptions noted in the acknowledgements. AP developed the concepts underpinning this work in discussion with KO-F, VP, and SS. NR and MM developed the simulants and methods to derive media composition, respectively. All authors contributed to development of the manuscript, which was primarily authored by AP.

## Funding

This work was supported by studentship funding from the Science and Technology Facilities Council and The Open University, as well as an Expanding Excellence in England (E3) grant from Research England (grant no. 124.18).

## Conflict of Interest

The authors declare that the research was conducted in the absence of any commercial or financial relationships that could be construed as a potential conflict of interest.

## Publisher’s Note

All claims expressed in this article are solely those of the authors and do not necessarily represent those of their affiliated organizations, or those of the publisher, the editors and the reviewers. Any product that may be evaluated in this article, or claim that may be made by its manufacturer, is not guaranteed or endorsed by the publisher.
