# Supplementary material for: Oligotrophic Growth of Nitrate-Dependent Fe2+-Oxidising Microorganisms Under Simulated Early Martian Conditions
Source: Front Microbiol. 2022 Mar 28;13:800219. doi: 10.3389/fmicb.2022.800219 (PMC8997339; doi:10.3389/fmicb.2022.800219)
Supplement: Supplementary file 1 [file Data_Sheet_1.docx]

Supplementary Material

Table S 1. Predicted ionic concentrations for fluids based on Contemporary Mars (CM), Sulfur-rich (SR), Haematite-rich (HR) and Shergottite (SG) simulants at a water-to-rock ratio (W/R) of 1000. Ions listed in descending order of predicted concentration for CM.

|  |  | Simulant | | | |
| --- | --- | --- | --- | --- | --- |
|  |  | CM | SR | HR | SG |
| Ion |  | Concentration (mM) | | | |
| Si | + | 7.351 | 3.602 | 6.828 | 8.585 |
| Fe^2+^ | + | 2.618 | 2.880 | 1.963 | 2.254 |
| Mg | + | 1.520 | 1.323 | 1.724 | 2.556 |
| Ca | + | 1.382 | 1.511 | 1.020 | 1.692 |
| NO_3_ | - | 1.000 | 1.000 | 1.000 | 1.000 |
| Al_2_ | + | 0.989 | 0.495 | 0.700 | 0.635 |
| Na_2_ | + | 0.542 | 0.309 | 0.373 | 0.264 |
| S | - | 0.466 | 3.649 | 0.534 | 0.050 |
| K_2_ | + | 0.253 | 0.164 | 0.171 | 0.079 |
| Fe^3+^_2_ | + | 0.110 | 0.105 | 0.961 | 0.076 |
| Ti | + | 0.092 | 0.058 | 0.062 | 0.032 |
| P_2_ | - | 0.040 | 0.210 | 0.036 | 0.040 |
| Mn | + | 0.034 | 0.016 | 0.023 | 0.028 |
| Cl | - | 0.029 | 0.021 | 0.023 | 0.020 |

Table S 2. Initial and final pH values for Mars simulant media cultures and abiotic controls (± standard error of triplicates).

| **Media** | **Inoculum** | **Initial pH** | **End point pH** |
| --- | --- | --- | --- |
| Contemporary Mars | *Paracoccus sp.* strain KS1 | 6.75 | 6.28 (±0.06) |
|  | *Pseudogulbenkiania sp.* strain 2002 | 6.75 | 6.39 (±0.07) |
|  | *Acidovorax sp.* strain BoFeN1 | 6.75 | 6.21 (±0.03) |
|  | Control | 6.75 | 6.28 (±0.07) |
| Sulfur-rich | *Paracoccus sp.* strain KS1 | 6.69 | 5.85 (±0.02) |
|  | *Pseudogulbenkiania sp.* strain 2002 | 6.69 | 5.86 (±0.02) |
|  | *Acidovorax sp.* strain BoFeN1 | 6.69 | 5.76 (±0.05) |
|  | Control | 6.69 | 5.79 (±0.01) |
| Haematite Slope | *Paracoccus sp.* strain KS1 | 6.72 | 6.36 (±0.03) |
|  | *Pseudogulbenkiania sp.* strain 2002 | 6.72 | 6.34 (±0.02) |
|  | *Acidovorax sp*. strain BoFeN1 | 6.72 | 6.30 (±0.04) |
|  | Control | 6.72 | 6.31 (±0.02) |
| Shergottite | *Paracoccus sp.* strain KS1 | 7.00 | 6.70 (±0.01) |
|  | *Pseudogulbenkiania sp.* strain 2002 | 7.00 | 6.74 (±0.06) |
|  | *Acidovorax sp.* strain BoFeN1 | 7.00 | 6.63 (±0.03) |
|  | Control | 7.00 | 6.67 (±0.07) |

Table S 3. Detection limits for ions detected by the Agilent 7500s ICP-MS system.

| Element | Detection limit (ppm) |
| --- | --- |
| Na | 3.21 |
| K | 2.02 |
| Ca | 0.14 |
| Si | 0.033 |
| Mg | 0.025 |
| ^56^Fe | 0.013 |
| ^57^Fe | 0.013 |
| Al | 0.013 |
| Sr | 0.0012 |
| Ba | 0.0007 |
| Mn | 0.0007 |

Table S 4. Correlation between protein concentration and nitrite production in inoculated cultures over 10 days.

| **Media** | **Organism** | **Correlation coefficient (*r*)** |
| --- | --- | --- |
| Contemporary Mars | *Paracoccus sp.* strain KS1 | 0.064766 |
|  | *Pseudogulbenkiania sp.* strain 2002 | 0.956911 |
|  | *Acidovorax sp*. strain BoFeN1 | 0.850712 |
| Shergottite | *Paracoccus sp.* strain KS1 | 0.855681 |
|  | *Pseudogulbenkiania sp.* strain 2002 | 0.955183 |
|  | *Acidovorax sp*. strain BoFeN1 | 0.972551 |

Table S 5. NPOC concentrations for non-starved (t=0) and starved (t=48 hours) inocula (± standard error of triplicates), with significance tested between the two groups.

|  |  | **Mean NPOC (mg l^-1^)** | |  |
| --- | --- | --- | --- | --- |
| **Variable** | | Non-starved | Starved | ***p* value** |
| **Media** | Contemporary Mars | 2.27 (± 0.36) | 3.36 (± 0.39) | 0.08 |
|  | Sulfur-rich | 0.65 (± 0.15) | 0.90 (± 0.18) | 0.23 |
|  | Haematite-rich | 1.04 (± 0.23) | 1.11 (± 0.22) | 0.78 |
|  | Shergottite | 1.20 (± 0.26) | 1.61 (± 0.45) | 0.28 |
|  | Minimal media | 0.44 (± 0.61) | 0.88 (± 0.18) | 0.53 |
| **Organism** | *Paracoccus sp.* strain KS1 | 0.40 (± 0.47) | 1.44 (± 0.40) | 0.06 |
|  | *Pseudogulbenkiania sp.* strain 2002 | 1.62 (± 0.26) | 2.34 (± 0.60) | 0.13 |
|  | *Acidovorax sp*. strain BoFeN1 | 1.41 (± 0.45) | 1.24 (± 0.52) | 0.14 |
|  | Control | 1.04 (± 0.30) | 1.27 (± 0.39) | 0.36 |


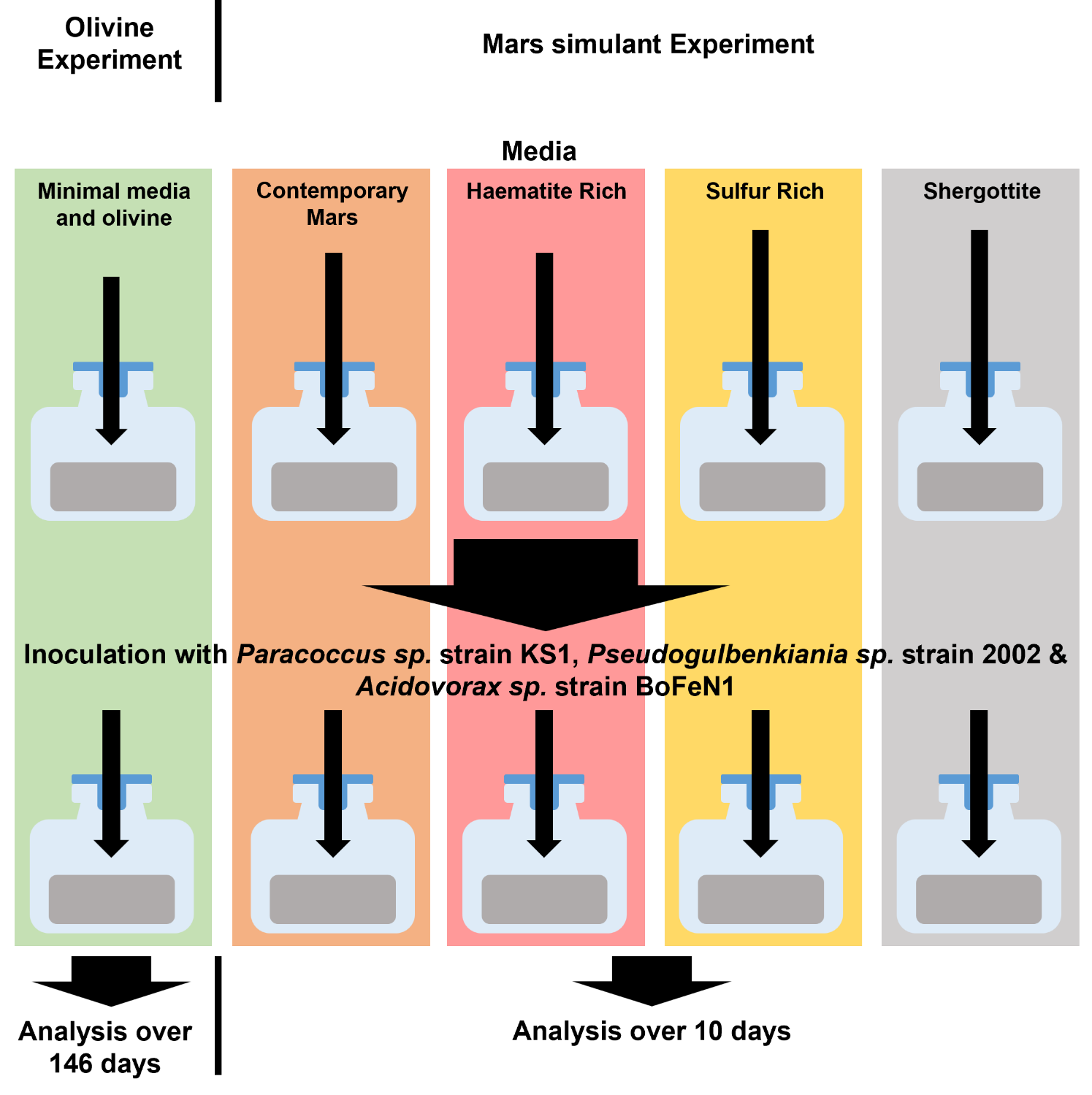


Figure S 1. Schematic diagram of the olivine and Mars simulant-derived media experiments.


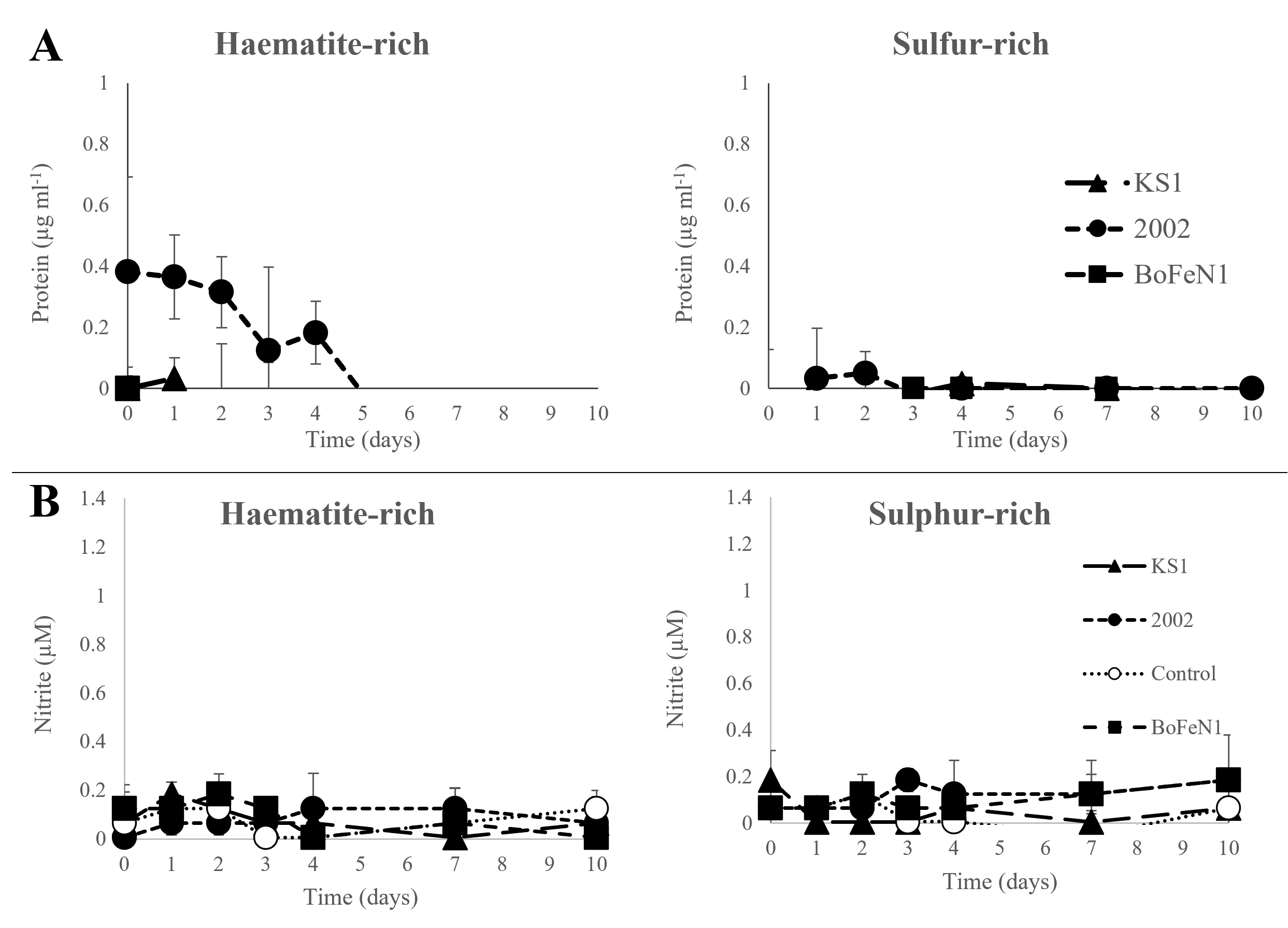


Figure S 2. (A) Protein and (B) nitrite concentration over time in the Haematite rich (HR) and Sulfur rich (SR) simulant media for *Paracoccus sp.* strain KS1, *Pseudogulbenkiania sp.* strain 2002, *Acidovorax sp.* strain BoFeN1 (± standard error of triplicates). Protein values are normalised against the abiotic control.


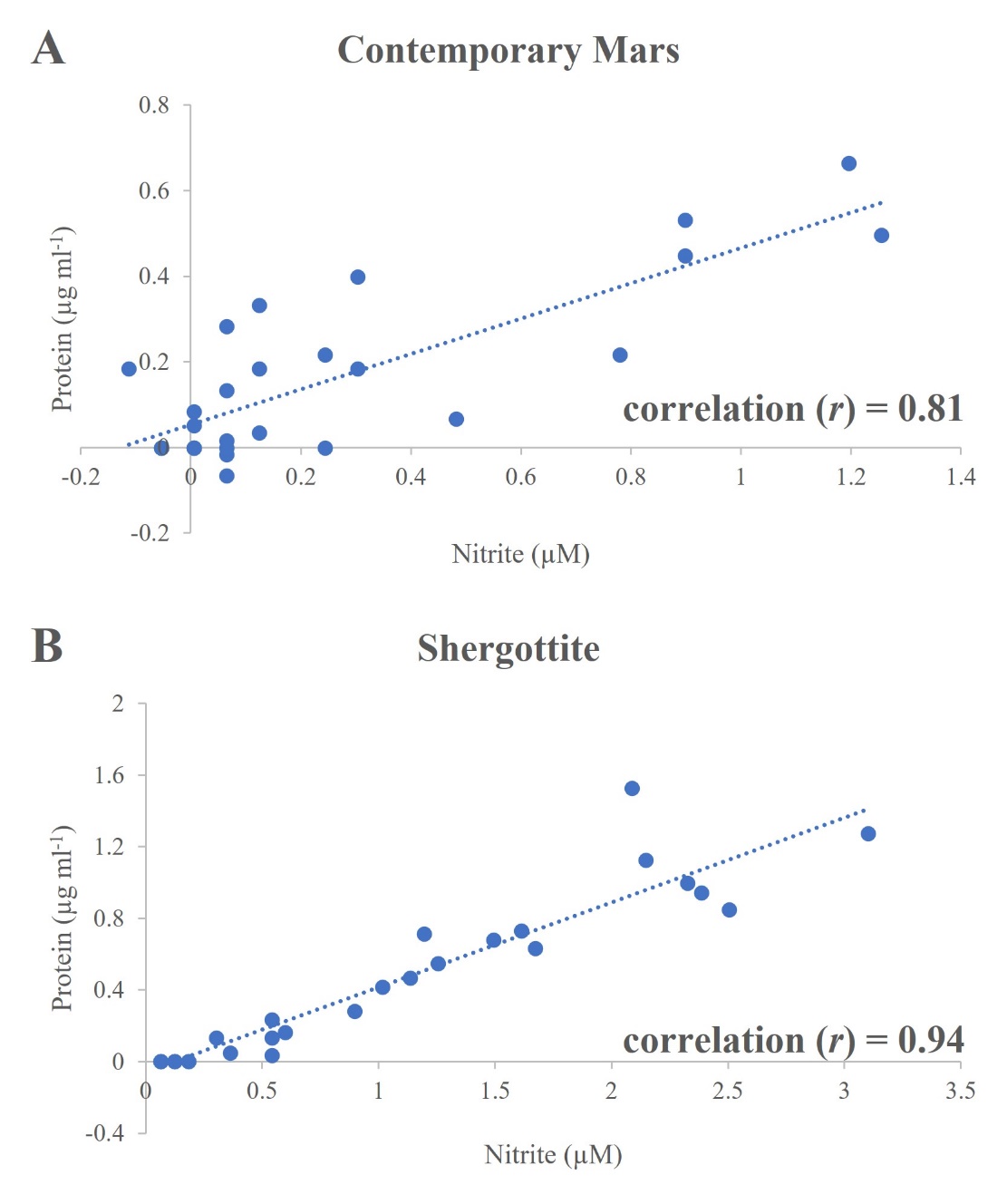


Figure S 3. Protein concentration vs nitrite concentration for all cultures and controls in (A) Contemporary Mars and (B) Shergottite simulant-derived media.


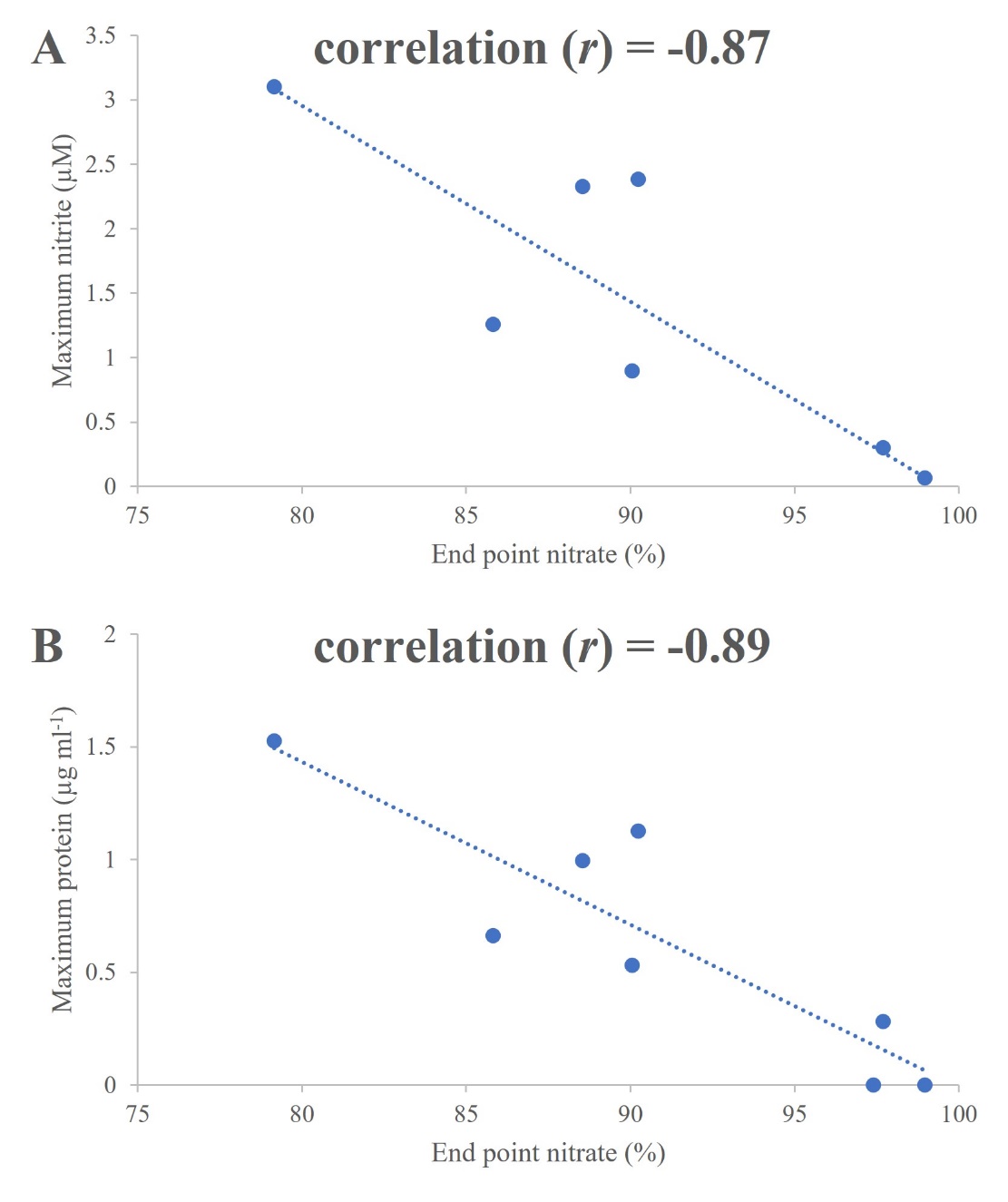


Figure S 4. (A) Maximum nitrite concentration and (B) maximum protein concentration vs end point nitrate concentration (as % of initial concentration). Data pooled from all cultures and controls in Contemporary Mars and Shergottite simulant-derived media.


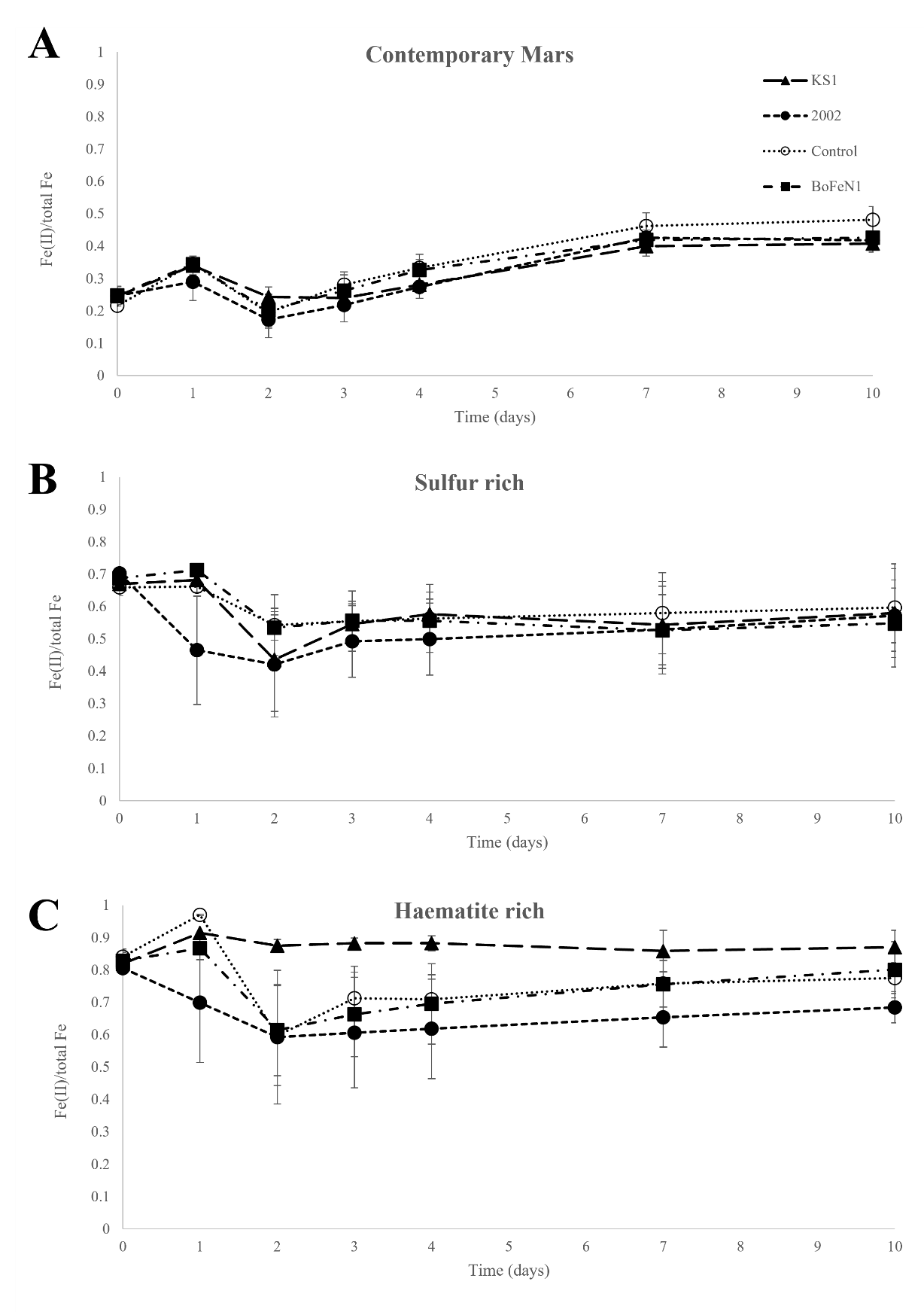


Figure S 5. Fe^2+^/Fe_total_ ratios for over time in the (A) contemporary Mars (CM), (B) Sulfur rich (SR) and (C) Haematite rich (HR) simulant media for *Paracoccus sp.* strain KS1, *Pseudogulbenkiania sp.* strain 2002, *Acidovorax sp.* strain BoFeN1 (± standard error of triplicates).


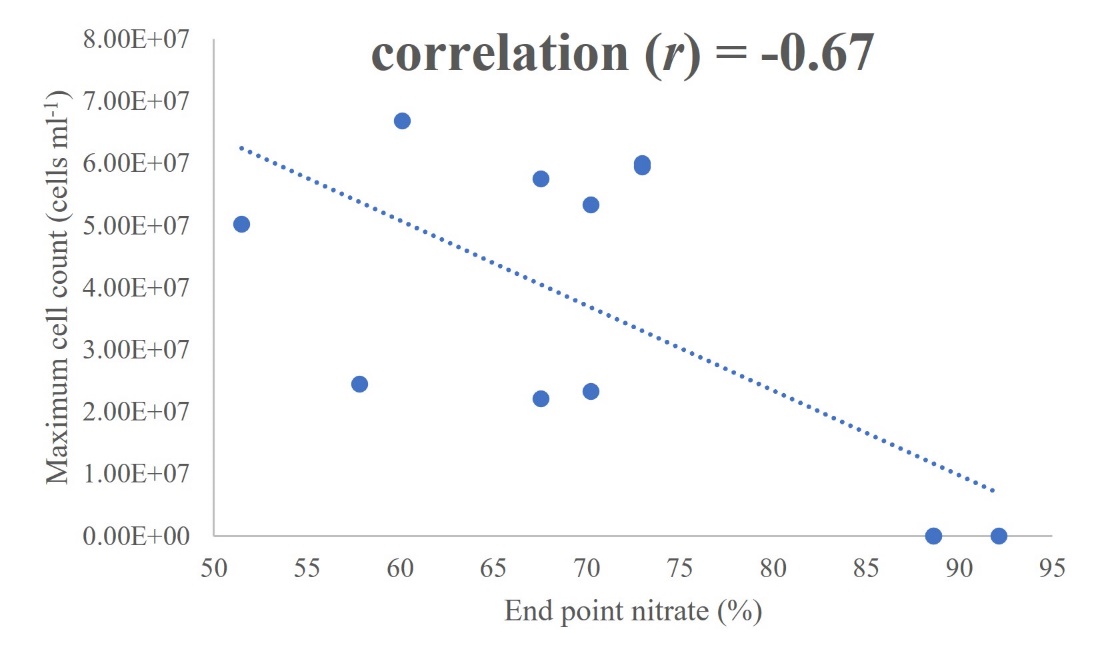


Figure S 6. Maximum cell density vs end point nitrate concentration (as % of initial concentration) for all cultures and controls in minimal media on olivine.


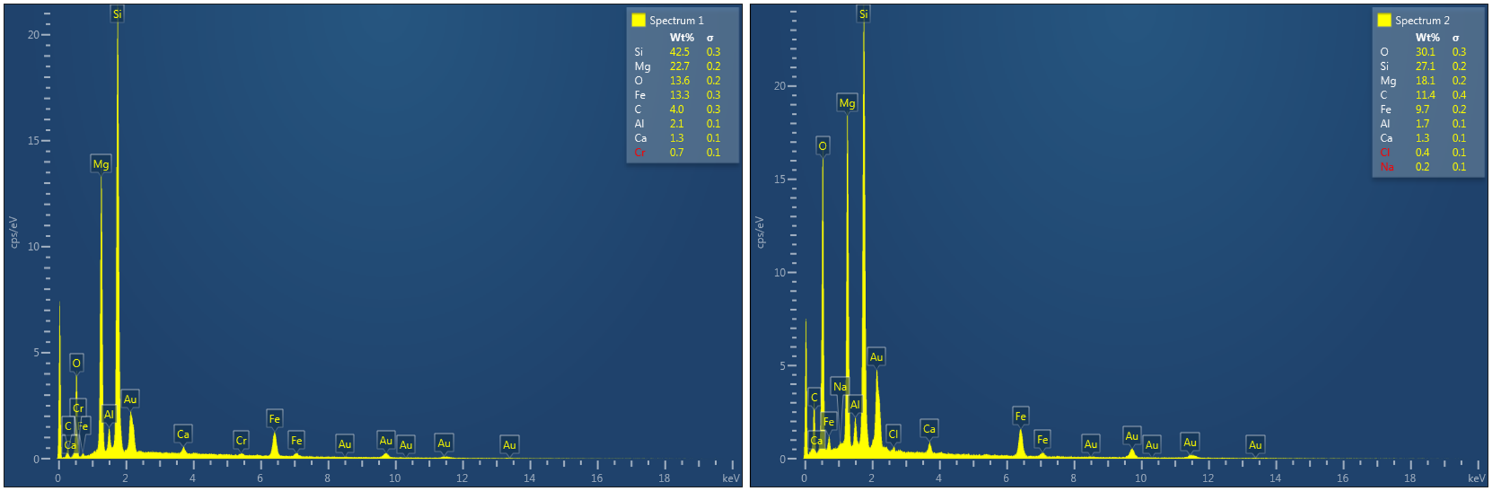


Figure S 7. Energy dispersive X-ray spectroscopy (EDS) elemental composition output for target area Spectra 1 and 2 indicated in Figure 3B.


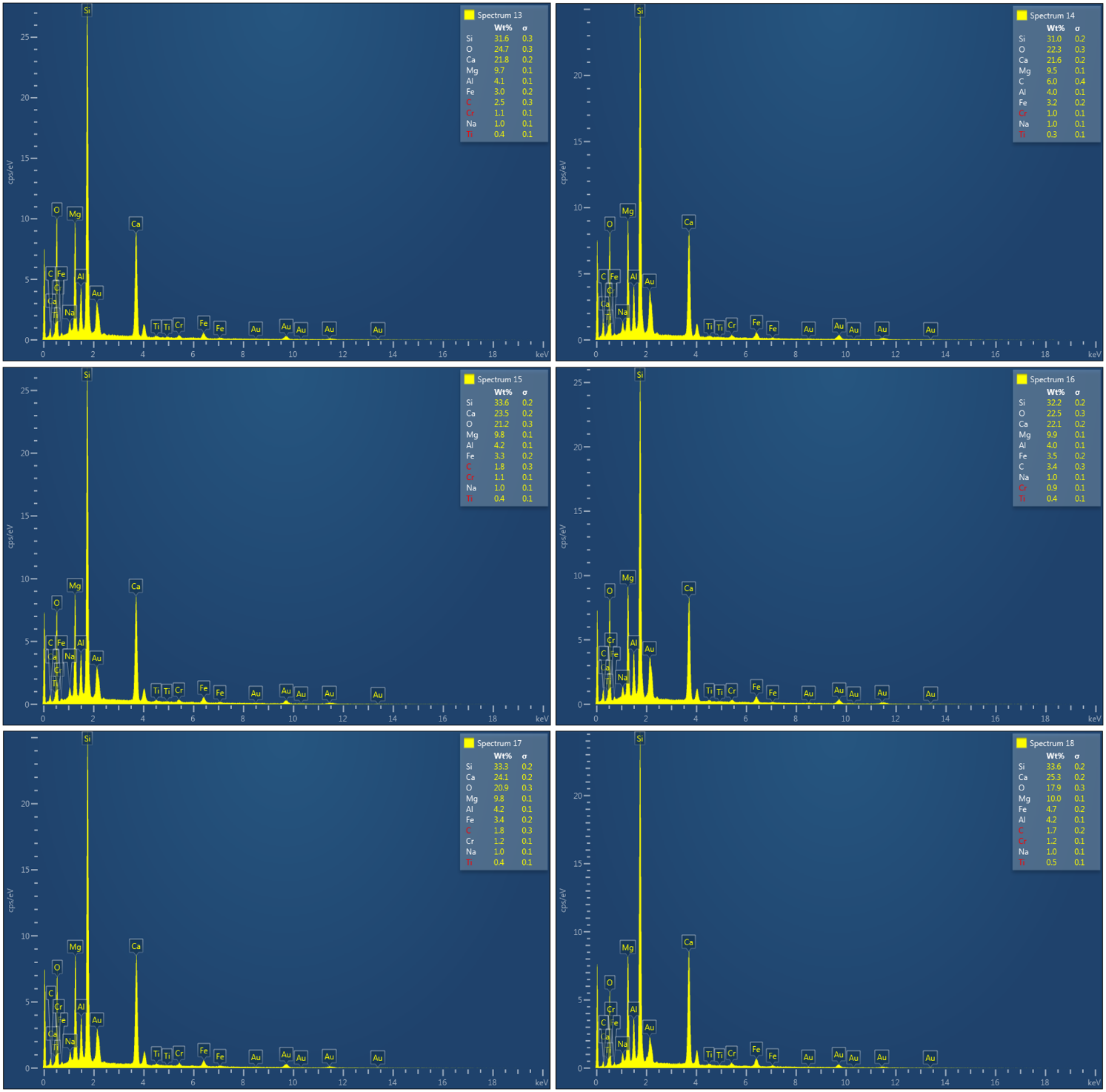


Figure S 8. Energy dispersive X-ray spectroscopy (EDS) elemental composition output for target area Spectra 13-18 indicated in Figure 3C.
